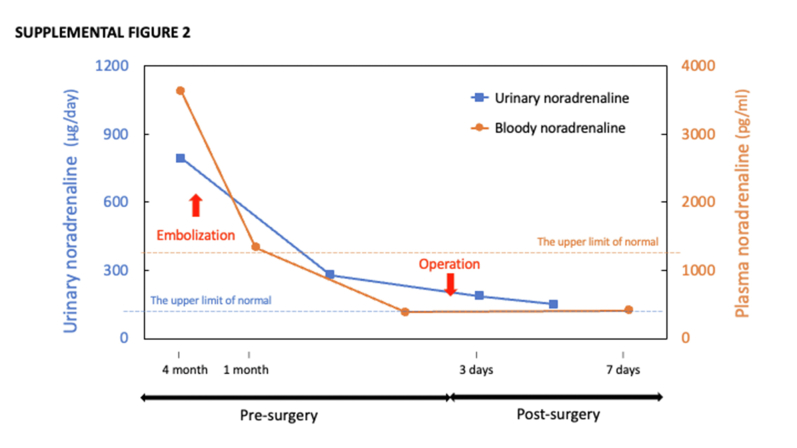# Successful Resection of Mediastinal Paraganglioma in an Adult After a Fontan Procedure

**DOI:** 10.1016/j.atssr.2025.01.007

**Published:** 2025-02-05

**Authors:** Ryo Sakada, Takaki Akamine, Sayaka Osawa, Hiromichi Sonoda, Fumihiko Kinoshita, Mikihiro Kohno, Keigo Ozono, Akira Shiose, Tomoyoshi Takenaka, Tomoharu Yoshizumi

**Affiliations:** 1 Department of Surgery and Science, Graduate School of Medical Sciences, Kyushu University, Fukuoka, Japan; 2 Department of Thoracic Surgery, Kyushu University Hospital, Fukuoka, Japan; 3 Department of Anesthesiology and Critical Care Medicine, Kyushu University Hospital, Fukuoka, Japan; 4 Department of Cardiovascular Surgery, Kyushu University Hospital, Fukuoka, Japan

## Abstract

Paraganglioma is a rare neuroendocrine tumor commonly located in the abdomen, head, and neck but rarely in the mediastinum. An association between the Fontan procedure and paragangliomas has been suggested. We report the case of a 28-year-old female patient incidentally diagnosed with a mediastinal paraganglioma at hypertension evaluation during cardiac catheterization post-Fontan procedure. We performed a preoperative right pulmonary artery occlusion test to ensure safe 1-lung ventilation. The tumor’s feeding vessels were embolized to minimize intraoperative blood loss. This case demonstrates successful resection of a mediastinal paraganglioma in a patient with Fontan circulation, using careful preoperative preparation and tumor embolization.

Paragangliomas are rare neuroendocrine tumors originating from extra-adrenal paraganglia, with an estimated incidence of 0.3 to 1 per 10,000 persons.^1^ These tumors occur in various locations, including the abdomen, head and neck, and thoracic cavity. Intrathoracic paragangliomas are infrequent (1%-2% of paragangliomas), arising mostly in the sympathetic chains in the posterior mediastinum.^1^

Paraganglioma is associated with the Fontan procedure, with an estimated prevalence of 2.5% in post-Fontan patients.^2^^,^^3^ Surgical treatment is the first choice for a mediastinal paraganglioma; however, 1-lung ventilation in post-Fontan patients is challenging because of increased pulmonary vascular resistance and impaired systemic venous return, which can cause circulatory failure.^4^ We report a case of mediastinal paraganglioma successfully resected in an adult patient after a Fontan procedure, achieved by careful preoperative management.

A 28-year-old female patient had undergone Glenn and Fontan procedures, along with previous operations for scoliosis and a duplicated esophagus (Supplementary Figure 1). Severe hypertension (systolic blood pressure, 200 mm Hg) had developed during routine cardiac catheterization after the Glenn and Fontan procedures. Further investigation using chest computed tomography revealed a 26-mm hypervascular mass on the aorta’s left side at the midthoracic esophageal level (Figure panel A). No masses were observed in the adrenal glands.

**Figure 1:**
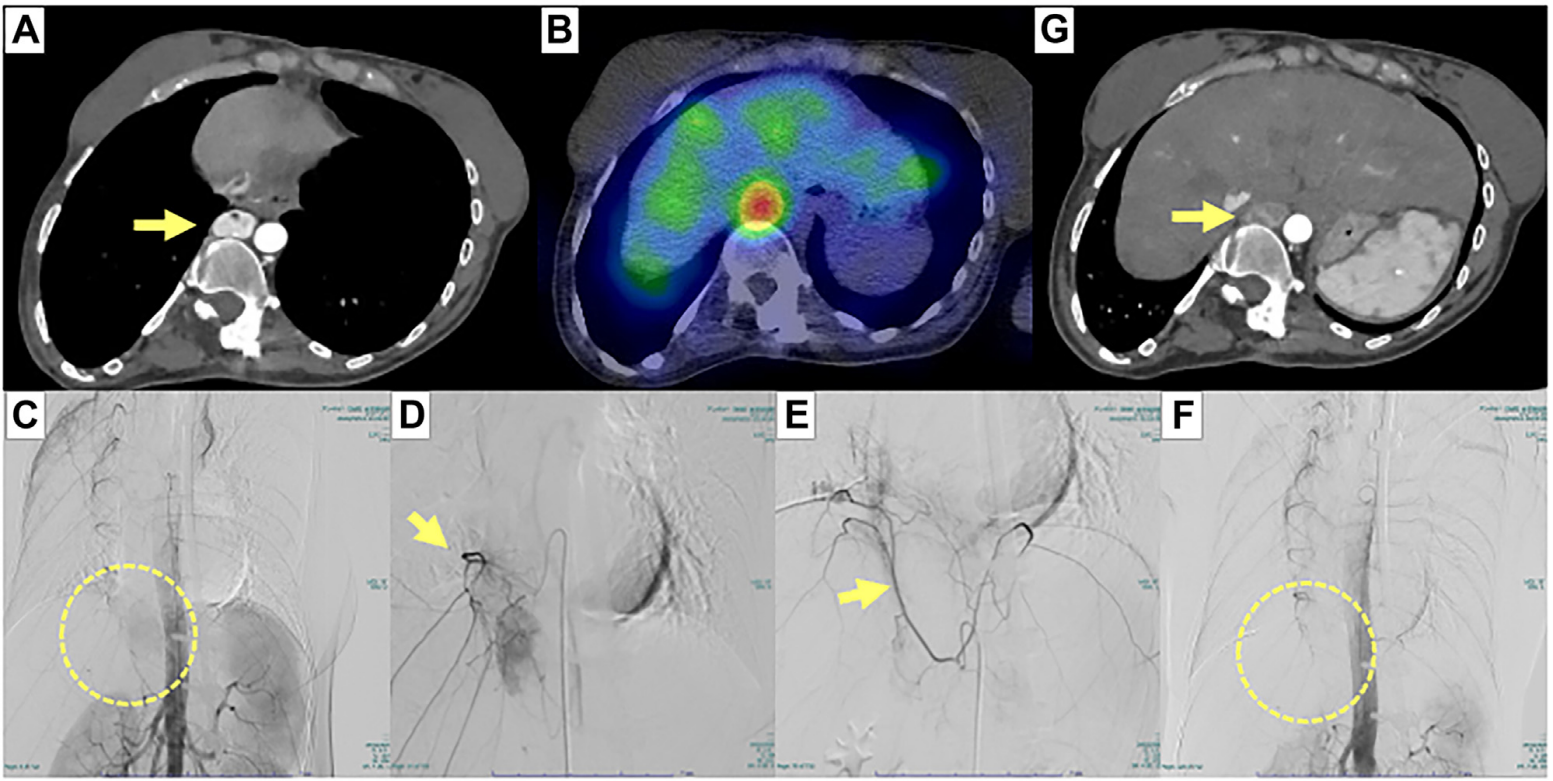
Preoperative embolization of a primary paraganglioma in the mediastinum. (A) Computed tomography (CT) shows paraganglioma primarily in the mediastinum before embolization (yellow arrowhead). (B) ^123^I-metaiodeobenzylguanidine shows abnormal accumulation (maximum standardized uptake value) in the same region. (C) Preoperative embolization is performed for a paraganglioma in the mediastinum. A catheter is inserted into the right 10th intercostal artery, and contrast is imaged (yellow circle). (D) Embolization is performed on the right 10th intercostal artery with embolic material (yellow arrowhead). (E) The right subdiaphragmatic artery is contrasted and embolized with a metal coil and embolic material (yellow arrowhead). (F) The tumor’s loss of contrast is evident after embolizing the tumor’s feeding arteries (yellow circle). (G) CT shows the contrast effect inside the mass is weakened (yellow arrowhead) after the embolization.

A ^123^I-metaiodeobenzylguanidine scan showed focal high tracer uptake in the tumor (Figure panel B). Biochemical analysis of the patient’s urine and blood showed elevated noradrenaline levels (Supplementary Figure 2), consistent with a diagnosis of a catecholamine-producing functional paraganglioma in the mediastinum.

The patient was prepared for surgery with oral administration of the α-adrenergic blocker doxazosin. Preoperative embolization of the tumor’s feeding arteries (the intercostal and right inferior phrenic arteries) was performed 2 months before surgery to minimize intraoperative blood loss (Figure panels C-F). A follow-up computed tomographic scan 1 month after embolization showed no change in tumor size, but the internal contrast effect had decreased (Figure panel G). In addition, a right pulmonary artery embolization test was performed using a 7F balloon wedge-pressure catheter to ensure the patient’s tolerance to 1-lung ventilation by confirming stable blood pressure and no increase in central venous pressure.

Intraoperative manipulation was expected to be difficult considering the patient’s previous posterior thoracotomy for resecting an esophageal duplication cyst, the presence of hepatomegaly (Fontan-associated liver disease), and scoliosis (Supplementary Figure 1).^5^ The patient was placed in a semilateral position under general anesthesia with 1-lung ventilation to confirm the absence of circulatory compromise. A cannula for venoarterial extracorporeal membrane oxygenation was placed through the right femoral artery and vein to manage circulatory failure owing to intraoperative hypertension during tumor manipulation.

An open lateral thoracotomy was performed through the eighth intercostal space. The tumor was in the middle of the mediastinum. Intraoperative manipulation caused a slight increase in blood pressure; however, no significant hypertension was observed. We identified and excised the arteries feeding from the descending aorta. After tumor removal, systolic blood pressure decreased slightly, but noradrenaline was not required. The operation was completed without circulatory compromise. The operative time was 1 hour 59 minutes with minimal blood loss (1 mL).

Postoperatively, the patient was stable without elevated blood pressure and α-blockers, and urinary catecholamine levels were within normal limits, indicating that the resection had been performed appropriately. The patient’s recovery was uneventful; she was discharged 9 days after surgery. Postoperatively, the blood norepinephrine and urinary adrenaline levels normalized.

## Comment

Mediastinal primary paragangliomas are extremely rare, especially in patients with a history of Fontan procedure. Here we present details of the surgical management of such rare tumors after the Fontan procedure. Considering the increasing survival rates of adult Fontan patients, the likelihood of encountering similar cases is expected to increase. This case report emphasizes the successful surgical resection of a mediastinal primary paraganglioma in a post-Fontan patient. We achieved this success from 2 perspectives: preoperative embolization and perioperative management.

Preoperative embolization for carotid body paraganglioma minimizes blood loss and shortens operative time; it is typically performed 1 to 7 days before surgery.^6^ In this case, however, embolization was performed 2 months before surgery without any complications, and the tumor was expected to shrink. Although its size remained unchanged, the tumor’s reduced contrast enhancement indicated the procedure was effective. During surgery, embolization was effective, minimizing blood loss and stabilizing intraoperative hemodynamics, suggesting that tumor embolization is an important strategy for managing thoracic paragangliomas.

Perioperative management of patients with Fontan disease presents a significant challenge because of the necessity for 1-lung ventilation, which increases pulmonary vascular resistance and compromises systemic venous return. These factors are especially important for patients with Fontan physiology.

There is currently no consensus regarding whether a preoperative pulmonary artery embolization test is needed for post-Fontan patients. After a multidisciplinary preoperative conference, we decided to perform this test owing to the high risks associated with this case, which included a history of right thoracotomy performed twice and a paraganglioma that could cause potential intraoperative blood pressure fluctuations. A similar case has been reported in which the preoperative embolization test was used to evaluate a post-Fontan patient's capacity to undergo 1-sided ventilation during thoracic surgery.^7^

Moreover, to manage potential intraoperative circulatory failure owing to hypertension during tumor manipulation, we prepared extracorporeal life support. Anesthetic management was carefully monitored to maintain low pulmonary vascular resistance and avoid hemodynamic instability.^4^^,^^8^

Integrating these strategies enabled the successful completion of the operation without hemodynamic compromise. This case demonstrates the utility of preoperative embolization and comprehensive perioperative planning for the effective surgical management of mediastinal paragangliomas in post-Fontan patients.

## Figures and Tables

**Supplementary Figure 1:**
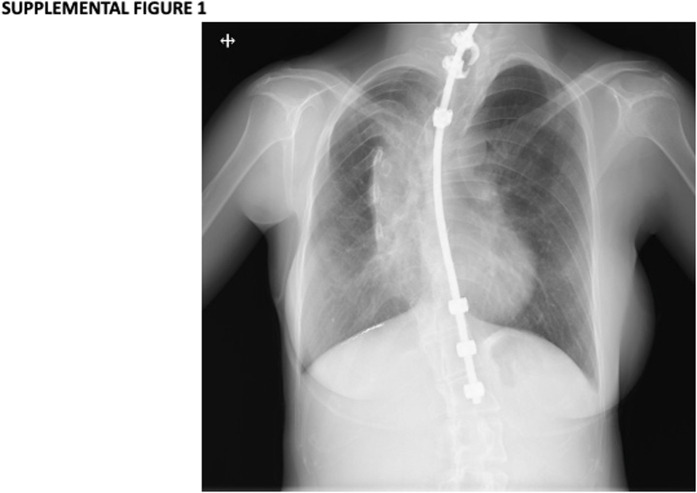


**Supplementary Figure 2:**