# Supplementary material for: Successful Resection of Mediastinal Paraganglioma in an Adult After a Fontan Procedure
Source: Ann Thorac Surg Short Rep. 2025 Feb 5;3(3):754–6. doi: 10.1016/j.atssr.2025.01.007 (PMC12559257; doi:10.1016/j.atssr.2025.01.007)
Supplement: Supplementary Figure legends [file mmc1.docx]

**SUPPLEMENTAL FIGURES**

**Figure 1.**

Chest x-ray shows scoliosis in the patient following spinal fusion.

**Figure 2.**

The biochemical analysis of the patient’s urine and plasma reveals elevated noradrenaline levels, consistent with a diagnosis of catecholamine production. The postoperative urinary and plasma noradrenaline levels are within normal limits.
